# Non-invasive methods to evaluate liver fibrosis in patients with non-alcoholic fatty liver disease

**DOI:** 10.3389/fphys.2022.1046497

**Published:** 2022-12-14

**Authors:** Jincheng Wang, Tao Qin, Jinyu Sun, Shiwu Li, Lihua Cao, Xiaojie Lu

**Affiliations:** ^1^ The First Affiliated Hospital of Nanjing Medical University, Nanjing, China; ^2^ Liver Disease Center, Qinhuangdao Third Hospital, Qinhuangdao, China

**Keywords:** NAFLD, non-invasive diagnosis, liver fibrosis, biomarkers, prediction

## Abstract

Non-alcoholic Fatty Liver Disease (NAFLD) is a chronic liver disease that is strongly related to insulin resistance and metabolic syndrome, and it has become the most common liver disorder in developed countries. NAFLD embraces the full pathological process of three conditions: steatosis, non-alcoholic steatohepatitis, and finally, cirrhosis. As NAFLD progresses, symptoms will become increasingly severe as fibrosis develops. Therefore, evaluating the fibrosis stage is crucial for patients with NAFLD. A liver biopsy is currently considered the gold standard for staging fibrosis. However, due to the limitations of liver biopsy, non-invasive alternatives were extensively studied and validated in patients with NAFLD. The advantages of non-invasive methods include their high safety and convenience compared with other invasive approaches. This review introduces the non-invasive methods, summarizes their benefits and limitations, and assesses their diagnostic performance for NAFLD-induced fibrosis.

## Introduction

Non-alcoholic Fatty Liver Disease (NAFLD) has become a significant cause of chronic liver disease worldwide (Loomba and Sanyal, 2013). For those patients with NAFLD, the critical issue is how to evaluate the stage of their diseases, as they face a significant risk of developing chronic liver diseases such as hepatocellular carcinoma or cirrhosis (Marignani and Angeletti, 2002; Byrne and Targher, 2015; Fan et al., 2017). The prognosis and management of NAFLD greatly depend on the progression of non-alcoholic steatohepatitis and liver fibrosis. Early-stage fibrosis is reversible, and patients will recover better if they get treatments in time.

Liver biopsy remains the most appropriate method for differentiating non-alcoholic fatty liver (NAFL) from non-alcoholic steatohepatitis and staging liver fibrosis (Byrne and Targher, 2015). However, its accuracy has been questioned, as liver biopsy presents some limitations, including sampling errors, variability, and invasiveness (Ratziu et al., 2005; Merriman et al., 2006; Bonekamp et al., 2014). These limitations contribute to the search for new non-invasive approaches to detect clinically significant samples and help patients get treatments ahead of time. Recent research is performed from two perspectives: serum biomarkers and imaging techniques (ultrasound, CT, MRI, *etc.*) for evaluating liver stiffness (Castera and Pinzani, 2010). We review non-invasive diagnostic methods for liver fibrosis and assess their advantages, limitations, and diagnostic performance in patients with NAFLD.

## Histologic stages of hepatic fibrosis

Patients with NAFLD suffer from a continuous spectrum of steatosis, inflammation, and fibrosis. Therefore, it would be difficult to assess the stage and progression of the disease. The SAF scoring system, developed by the European consortium for Fatty Liver Inhibition of Progression, was specifically designed to evaluate NALFD. For each case, a SAF score was created based on the semiquantitative scoring of steatoses (S), activity (A), and fibrosis (F). The stage of fibrosis (F) was assessed using the score described by non-alcoholic steatohepatitis-CRN as follows: stage 0 (F0) none; stage 1 (F1): 1a or 1b perisinusoidal zone 3 or 1c portal fibrosis; stage 2 (F2): perisinusoidal and periportal fibrosis without bridging; stage 3 (F3): bridging fibrosis; and stage 4 (F4): cirrhosis (Bedossa and Consortium, 2014).

## Diagnosis and staging of hepatic fibrosis

Hepatic fibrosis can be non-invasively measured through two complementary approaches, including a “biological” approach (quantifying serum biomarkers) or a “physical” approach (measuring liver stiffness with the use of image technology). These two approaches can perform their unique functions according to different rationales. Imaging-based liver stiffness is consistent with an intrinsic physical property of liver tissue (European Association for Study of, 2015).

## Serum biomarkers

Serum biomarkers can be divided into direct markers and indirect markers. Direct markers can reflect the deposition or removal of fibrotic tissue in the liver. Indirect markers are markers of comprehensive liver function.

## Routine laboratory tests

Indirect markers, such as serum bilirubin and albumin levels, are often abnormal in patients with cirrhosis, and prothrombin time will increase (Schuppan and Afdhal, 2008). The platelet count will be low because of the hypersplenism related to portal hypertension. When these laboratory markers become abnormal, liver fibrosis is often already clinically apparent and irreversible. Although these biomarkers might help evaluate the stage of advanced liver diseases, they are often not able to detect early-stage fibrosis. Other indirect markers include the ratio of aspartate aminotransferase to alanine aminotransferase (AST/ALT), AST: platelet ratio index (APRI), *α*_2_-macroglobulin (A2M), apolipoprotein A1, glutamyl transpeptidase (GGT). The connection between AST/ALT ratio and liver fibrosis has been confirmed (Sheth et al., 1998). The ratio of AST/ALT is often <1 in patients with early-stage fibrosis (F1-F2). However, it will increase with the stage of fibrosis evolving into cirrhosis (Angulo et al., 1999). The APRI score (AST/platelet ratio) is recommended as another marker for advanced liver fibrosis. The accuracy of the APRI score in assessing the stage of liver fibrosis in patients with NAFLD has been confirmed in many studies (Kruger et al., 2011). A study involving 111 patients reported an AUROC value of APRI of 0.85, a Se of 75%, an Sp of 86%, a PPV of 54%, and an NPV of 93%. Elevated serum ferritin levels have been found in patients with NAFLD (Chitturi et al., 2002; Bugianesi et al., 2004). Most researchers believe high serum iron indices are related to liver damage and inflammation (Chitturi et al., 2002; Bugianesi et al., 2004; Manousou et al., 2011; Feldman et al., 2016). A recent cross-sectional descriptive study involving 284 patients confirmed a significant connection between serum ferritin levels and liver stiffness. Therefore, a low serum ferritin level may be one cost-effective option to exclude patients with advanced fibrosis from liver biopsy and elastography (Seyedian et al., 2017).

In recent years, novel serum biomarkers have been discovered, including Mac-2 binding protein glycan isomer (M2BPGi), *Wisteria floribunda* agglutinin-positive Mac-2 binding protein (WF), soluble Axl (sAxl), osteopontin, angiotensin-converting enzyme (ACE) (Pereira et al., 2016; Miranda and Simoes, 2017; Staufer et al., 2017; Ogawa et al., 2018; Shirabe et al., 2018), and cytokeratin 18 (M30 and M65) (Lee et al., 2020). However, the accuracy of these biomarkers as a marker of fibrosis is still unknown. More research is needed before they can be recommended as appropriate fibrosis severity markers.

## Combination with clinical features

To improve the sensitivity and predictive effect of simple laboratory tests for liver fibrosis, serum biomarkers (most direct markers and several indirect markers) and clinical features have been combined to create several multivariate analyses for liver fibrosis: the FIB-4 index; the NAFLD fibrosis score; the ELF (Siemens Healthcare, Erlangen, Germany); the BARD score; the FibroTest (BioPredictive, Paris, France); the FibroMeter™ NAFLD (BioPredictive, Paris, France); and the Hepascore (Quest Diagnostics, Madison, NJ) (Table 1) (Ratziu et al., 2006; Guha et al., 2008; Cales et al., 2009; Nobili et al., 2009; Shah et al., 2009; Adams et al., 2011; Musso et al., 2011; Sumida et al., 2012; Dincses and Yilmaz, 2015).

**TABLE 1:** Performance of selected serum biomarkers in patients with NAFLD.

Score	Patients	Predictive variables	End points	Cut-offs value	AUROC	Se	Sp
NAFLD	N = 3064 NAFLD from 13 studies	Age, fasting glucose	F ≥ 3	<-1.455	0.85	0.90	0.60
Fibrosis score		BMI, platelets, Albumin, AST/ALT		≥0.676		0.64	0.97
BARD	N = 1506 NAFLD from 6 studies	BMI≥28, AST/ALT≥0.8 diabetes	F ≥ 3	2	0.78	0.72	0.64
ELF(Siemens Healthcare, Erlangen, Germany)	N = 192 NAFLD	HA, PIIINP, TIMP-1	F ≥ 2	-0.1068	0.82	0.70	0.80
			F ≥ 3	0.3576	0.90	0.80	0.90
Fibro Meter (Echosens, Paris, France)NAFLD	N = 235 NAFLD	Age, weight, AST, ALT, ferritin, glucose, platelets	F ≥ 2	≤0.611	0.94	0.79	0.96
				≥0.715			
Fibro Test (BioPredictive, Paris, France)	N = 267 NAFLD	A2M, APOA1, GGT, haptoglobin, total bilirubin	F ≥ 2	0.30	0.81	0.77	0.77
	N = 954 controls		F ≥ 3	0.70	0.88	0.15	0.98
				0.30		0.92	0.71
				0.70		0.25	0.97
FIB-4	N = 541NAFLD	Age, AST, ALT platelets	F ≥ 3	<1.30	0.80	0.74	0.71
				>2.67		0.33	0.98
Hepascore (Quest Diagnostics, Madison, NJ)	N = 242NAFLD	Age, sex, A2M, HA, bilirubin, GGT	F ≥ 2	0.44	0.73	0.50	0.88
			F ≥ 3	0.37	0.81	0.75	0.84
			F4	0.70	0.91	0.87	0.89

ALT, alanine aminotransferase; APOA1, apolipoprotein A-1; AST, aspartate aminotransferase; AUC, area under the concentration-time curve; A2M, a-2 macroglobulin; ELF, enhanced liver fibrosis; GGT, g-glutamyltranspeptidase; HA, hyaluronic acid; PIIINP, N-terminal propeptide of type III, collagen; TIMP-1, tissue inhibitor of metalloproteinase 1.

A simple scoring system such as the NAFLD fibrosis score could accurately separate patients with NAFLD with and without advanced fibrosis and allow a substantial proportion of patients to avoid liver biopsies (Angulo et al., 2007). In a meta-analysis of 3064 people with NAFLD, the value of the Area Under the Receiver Operating Characteristic (AUROC) for patients with stage 3–4 fibrosis was 0.85^26^The NAFLD practice guidelines emphasized its effectiveness in identifying patients with advanced NAFLD (Chalasani et al., 2012). The American Association for the Study of Liver Disease has endorsed its use to routinely determine the need for liver biopsy for fibrosis staging in patients with NAFLD.

FIB-4 has been validated independently by three NAFLD cohorts in the United States, Europe, and Asia. Although these studies have used different cutoff values, they were successful in excluding advanced fibrosis (Shah et al., 2009; McPherson et al., 2010; Sumida et al., 2012). The ALT/AST ratio, FIB-4, and NAFLD fibrosis scores can reliably exclude advanced fibrosis in a high proportion of patients with NAFLD, allowing liver biopsy to be used in a more directed manner.

Complex fibrosis models such as Hepascore, FibroTest, and FIB4 have been reported to be more accurate in detecting fibrosis than simple fibrosis models (BARD or APRI) (Adams et al., 2011; Musso et al., 2011). When compared with the NAFLD fibrosis score, these biomarker panels generally have comparable accuracy in diagnosing advanced liver fibrosis in patients with NAFLD (Cales et al., 2009).

The FibroMeter NAFLD, which estimates the stage of fibrosis based on age, weight, AST, ALT, ferritin, glucose, and platelets values, has been validated in a NAFLD cohort with 235 patients. The AUROC to detect advanced fibrosis (F3-F4) was excellent and markedly better than the APRI (Cales et al., 2009).

The Enhanced Liver Fibrosis (ELF) test is a commercially available algorithm that includes three serum biomarkers: hyaluronic acid (HA), the N-terminal pro-peptide of collagen type III (PIIINP), and tissue inhibitor of metalloproteinase-1 (TIMP1) (Rosenberg et al., 2004; Guha et al., 2008). The ELF (the cutoff value is 10.5) was recently recommended to detect advanced fibrosis in patients with NAFLD (Glen et al., 2016). However, this recommendation is contentious because it is based on a pediatric NAFLD study (Nobili et al., 2009). A recent prospective, direct comparison of tests that included 289 patients showed that the AUROC of ELF-identified patients with advanced liver fibrosis is 0.92 (95% confidence interval 0.89–0.96). This study also compared ELF with FibroTest and Elastography. ELF had a generally high diagnostic accuracy (AUROC values of 0.90 or higher) in identifying advanced liver fibrosis (Thiele et al., 2018

All these algorithms and systems, which combine serum biomarkers and clinical features, seem helpful in identifying patients with a low risk of advanced liver fibrosis, so a liver biopsy for staging purposes can be avoided. However, none of these algorithms were designed to predict disease progression. Moreover, these models are not sufficiently accurate for patients with suspected advanced fibrosis to replace a liver biopsy.

## Imaging

### Transient elastography

Transient Elastography (TE; FibroScan, Echosens, Paris, France) is the imaging technique used most frequently to assess fibrosis in patients with NAFLD in clinical practice. The stage of liver fibrosis can be assessed using TE (Sandrin et al., 2003).

TE is a technique based on ultrasound (United Kingdom) (5 MHz) and low-frequency (50 Hz) elastic waves, whose propagation velocity through the liver is directly related to liver tissue stiffness. Elastic modulus is the terminology used to describe tissue stiffness and is expressed as E = 3 ρv (Marignani and Angeletti, 2002), where v is the shear velocity and *?* is the density of tissue as an invariant. In brief, the faster propagation of the shear wave indicates stiffer tissue. TE measures liver stiffness in a volume that is approximately a 1 cm wide per 4 cm long cylinder, with the M probe measuring 25–65 mm and the XL probe measuring 35–75 mm below the skin surface (Roulot et al., 2008). The values are expressed in kilopascals (kPa) which range between 2.4 and 75.4 kPa. The optimal LSM cutoff for maximum specificity and sensitivity ranges from 7.2 to 11.4 kpa (Wong et al., 2012; Imajo et al., 2016).

TE-based liver stiffness measurements using the M probe have been shown to correlate with fibrosis stages, particularly in severe fibrosis and cirrhosis (Friedrich-Rust et al., 2008; de Ledinghen et al., 2012; Pavlov et al., 2016). The benefits of using TE to measure liver stiffness include a quick procedure (<5 min), immediate results, and the ability to conduct the test when the patient is in the hospital or an outpatient clinic. It is easy to learn the procedure of TE and can be performed by a medical intern or a nurse after a bit of training (Tsai and Lee, 2018). However, accurate TE results require careful interpretation of data based on at least ten validated measurements, limiting its simplicity, application, and stability (Castera et al., 2008). An important limitation of TE is the high failure rates in overweight or obese patients with a BMI >28 kg/m^2^, which limits the measurement of steatosis and liver stiffness in obese patients with NAFLD (Foucher et al., 2006; de Ledinghen et al., 2014). However, a new TE probe (XL) equipped with CAP has been proposed to reduce the failure rate of detecting fibrosis in patients who are overweight or obese (de Ledinghen et al., 2012; Wong et al., 2012).

### Performance of TE for staging liver fibrosis

Although TE has been evaluated in many studies on patients with viral hepatitis, it has been used in fewer studies of NAFLD. TE estimated cirrhosis with higher AUROC values (0.95–0.97) than significant fibrosis (0.80) (Castera, 2015). A meta-analysis including nine studies and 1047 patients with NAFLD found that (vibration-controlled transient elastography) VCTE detected cirrhosis with an aggregate value of 92%, significant fibrosis with a 79% sensitivity, and cirrhosis with a 75% sensitivity (Kwok et al., 2014). Table 2 enumerates the diagnostic performance of TE for measuring advanced liver fibrosis in patients with NAFLD (Nobili et al., 2008; Yoneda et al., 2008; Lupsor et al., 2010; Wong et al., 2010; Gaia et al., 2011; Petta et al., 2011; Myers et al., 2012; Wong et al., 2012; Kumar et al., 2013; Mahadeva et al., 2013; Aykut et al., 2014; Naveau et al., 2014; Chan et al., 2015; Petta et al., 2015; Boursier et al., 2016; Cassinotto et al., 2016; Imajo et al., 2016; Pavlov et al., 2016; Tapper et al., 2016; Chen et al., 2017; Park et al., 2017).

**TABLE 2:** Performance of transient elastography in patients with NAFLD.

Authors	Year	Sample size	Mean BMI (kg/m2)	Failure rate (%)	F3-F4 (%)	Cut-offs value	AUROC	Se	Sp
Yoneda et al	2008	97	26.6	4.9	28	8.2	0.90	85	81
Nobili et al	2008	50	25.7	3.8	20	7	0.94	100	100
Wong et al	2010	246	28.5% > 30	10.2	23	8.7	0.93	84	83
Lupsor et al	2010	65	28.7	9.7	7	7.5	0.98	100	96
Petta et al	2011	146	29.1	14	14	8.75	0.87	76	78
Gaia et al	2011	72	27.5	8	34	8.0	0.76	65	80
Myers et al	2012	75	30.0	16(M)	28	7.8 (M)	0.87(M)	84(M)	79(M)
				1.1 (XL)		6.4 (XL)	0.90 (XL)	81 (XL)	66 (XL)
Wong et al	2012	193	28.9	25	30	7.2 (XL)	0.78(ITT) 0.85(PP)	74(ITT)/78(PP)	78(ITT)/78(PP)
Kumar et al	2013	120	26.1	14.9	23	9.0	0.94	85	88
Mahadeva et al	2013	131	32.8% > 30	8	22	7.1	0.77	70	67
Aykut et al	2014	88	30.3	NA	41	7.9	0.94	96	90
Naveau et al	2014	100	42.3	19	9	7.6	0.85	100	74
Petta et al	2015	324	39.5% > 30	NA	35	10.1	0.86	78	78
Chan et al	2015	153	29.4	3.9	21	8.0	NA	95	66
Cassinotto et al	2016	291	60.1% > 30	23.4	43	8.2/12.5	0.86	90/57	61/90
Imajo et al	2016	142	28.1	10.5	32	11.4	0.88	86	84
Tapper et al	2016	164	32.3	27	18	9.9	0.93	95	77
Boursier et al	2016	588	31.7	9.3	39	8.7	0.83	88	63
Chen et al	2017	111	40.3	22.7	20	7.6	0.87	84	64
Park et al	2017	104	30.4	6.7	17	7.3	0.8	78	78

AUROC, area under ROC, curve; CC, correctly classified: true positive and true negative; NAFLD, Non-alcoholic fatty liver disease; NA, not available; Se = sensitivity; Sp = specificity; ITT; intention to treat.

These studies indicate that TE could be used to confidently exclude severe fibrosis in NAFLD. Still, the high rate of unreliable results with transient elastography remains a challenge, which is not entirely addressed using the XL probe. However, due to the high prevalence of NAFLD in the general population, using TE could be significant in helping to determine which patients still require liver biopsies.

### Shear-wave elastography

Shear-Wave Elastography (SWE) is a novel, non-invasive method that the FDA has approved to assess liver stiffness. In SWE, the operator targets the liver using a 2D mode ultrasonography image to find a homogeneous area free of large vascular structures. The variable depth and diameter of the region of interest are defined in the visualized liver. The shear wave propagation speed in the area will be recorded by converting it into stiffness measurements, and a color map superimposed on the 2D-mode images can be constructed (Deffieux et al., 2015).

### Performance of SWE for staging liver fibrosis

Compared with TE, fewer studies focus on the performance of SWE. The largest study, including 291 patients with NAFLD, found that the combined failure rates were 20.3% and the AUROC values for advanced fibrosis were 0.89. Cutoffs of 8.7 kPa provided 90% sensitivity and specificity (Ochi et al., 2012; Cassinotto et al., 2016).

### Acoustic radiation force impulse imaging

With technological advances and clinical practice, Acoustic Radiation Force Impulse imaging (ARFI) is considered a standard ultrasound device. The ARFI operator, using a curved abdominal probe, defines a large area free of large vascular structures. Following that, short-duration (∼262 μs) acoustic pulses (with frequencies between 1.0 and 4.5 MHz) propagate shear waves and generate localized, *µ*-scale displacements in liver tissue. The ultrasound receiver tracks the shear-wave velocity of ARFI in a smaller volume (5 mm by 4 mm) than TE. The significant advantage of ARFI is that it can be easily implemented on a regular ultrasound machine. In addition, it has higher applicability than TE (Friedrich-Rust et al., 2009).

### Performance of ARFI for staging liver fibrosis

ARFI performance for staging liver fibrosis has been evaluated in five studies of patients with NAFLD (Table 3) (Ebinuma et al., 2011; Palmeri et al., 2011; Cassinotto et al., 2016; Cui et al., 2016). The AUROC for advanced fibrosis is 0.90, similar to TE and SWE. However, it also suffers from the risks of technical failure or unreliable results. In addition, technical failure rates tend to increase in patients with higher BMI (>30 kg/m^2^). Although the optimal cutoff is hard to determine, a shear-wave speed (about 1.34 m/s) is closely associated with advanced fibrosis (Ebinuma et al., 2011; Yoon et al., 2012). The largest study up to date by Cassinoto *et al.* provides cutoffs of 1.15 m/s and 1.53 m/s, which yield 90% sensitivity and specificity, respectively (Cassinotto et al., 2016).

**TABLE 3:** Performance of Shear-wave elastography and ARFI in Patients with NAFLD.

Authors	Year	Technique	Size	Mean BMI (kg/m^2^)	Failure rate (%)	F3-F4 (%)	Cut-offs value (kPa)	AUROC	Se	Sp
Ochi et al	2012	SWE	181	26.5	20.3	43	8.3 and 10.7	0.89	91/71	71/90
Cassinotto et al	2016	SWE	291	60.1% > 30	NA	28	3.02	0.88	89	97
Palmeri et al	2011	ARFI	172	68.6% > 30	21.5	33	2.06	0.9	90	90
Cassinotto et al	2016	ARFI	291	60.1% > 30	19	43	1.15	0.84	90	63
Cui. et al	2016	ARFI	125	31.8	2.4	17	1.34	0.9	95	74

AUROC, area under ROC, curve; CC, correctly classified: true positive and true negative; NAFLD, Non-alcoholic fatty liver disease; NA, not available; Se = sensitivity; Sp = specificity.

### Magnetic resonance elastography

Magnetic Resonance (MR) elastography has been shown to accurately diagnose fibrosis and cirrhosis in patients with NAFLD. MR elastography can be implemented on a conventional magnetic resonance imaging (MRI) system with special adaptation software. The propagation characteristics of the shear wave in the liver can be imaged with a modified phase-contrast method. Elasticity is quantified by MR elastography (expressed in kPa) using a formula that calculates the shear modules. MR elastography has obvious advantages compared with other imaging methods, including the assessment of almost the entire liver and a lower technical failure rate (Venkatesh et al., 2013; Loomba et al., 2014; Loomba et al., 2016). The largest study of MR elastography found that the failure rate was 7.7% in patients with NAFLD (Wagner et al., 2017). In general, MR elastography performs better than all ultrasound-based methods. Moreover, it has a lower risk of failure in patients with severe obesity (Cui et al., 2015; Cui et al., 2016).

### Performance of MRE for staging liver fibrosis

Six studies have focused on the performance of MR elastography for staging liver fibrosis (Table 4) (Huwart et al., 2008; Talwalkar, 2008; Loomba et al., 2014; Cui et al., 2015; Cui et al., 2016; Imajo et al., 2016; Loomba et al., 2016; Chen et al., 2017; Park et al., 2017). These studies found that MR elastography has a similar accuracy in diagnosing significant fibrosis and cirrhosis. In all studies, the AUROC values ranged between 0.92 and 0.94 for the diagnosis of significant fibrosis. However, the optimal cutoff values remained undetermined. In a study enrolling 100 patients with NAFLD (mean BMI of 32.4 kg/m^2^), the optimal cutoff for significant fibrosis and cirrhosis was 3.63 kPa. Another study, involving 104 patients, compared the performance of MR elastography and TE and found that the optimal cutoff value was 2.99 kPa (Park et al., 2017).

**TABLE 4:** Performance of MRE for in patients with NAFLD.

Authors	Year	Size	Mean BMI (kg/m2)	Failure rate (%)	F3-F4 (%)	Cut-offs value (kPa)	AUROC	Se	Sp
Loomba et al	2014	117	32.4	0	19	3.6	0.92	82	90
Cui et al	2015	102	31.7	0	19	3.6	0.96	92	90
Loomba et al	2016	100	32.1	0	15	3.8	0.92	80	95
Imajo et al	2016	142	28.1	0	32	4.8	0.89	75	87
Park et al	2017	104	30.4	0	17	3.0	0.87	78	80
Chen et al	2017	111	40.3	4.5	20	3.6	0.92	84	83

AUROC, area under ROC, curve; CC, correctly classified: true positive and true negative; NAFLD, Non-alcoholic fatty liver disease; NA, not available; Se = sensitivity; Sp = specificity.

### Comparison of approaches

Studies comparing TE and biomarkers, and other imaging methods, are limited. When patients had successful imaging-logic examinations, TE performed better than these simple laboratory serum biomarkers in detecting advanced fibrosis (Castera et al., 2005; Poynard et al., 2008; Adams, 2010; Wong et al., 2010; Kim et al., 2013a). However, signal-positive predictive results remain suspect because of some well-known confounders, including obesity and liver inflammation. This is the reason why strategies combining imaging approaches and serum biomarkers have shown increased diagnostic accuracy. Moreover, positive values need careful evaluation considering factors such as age, laboratory tests, and the scoring system (the NAFLD score, FIB-4).

A study of 291 patients comparing TE with ARFI and SWE found that no method could get more reliable results than others (Cassinotto et al., 2016). They also shared similar AUROC values for advanced fibrosis (VCTE 0.86, SWE 0.89, and ARFI 0.84). Three studies compared TE with MR elastography (Cui et al., 2016; Imajo et al., 2016; Park et al., 2017). An analysis of 143 patients has compared MR elastography with simple algorithms such as the NAFLD fibrosis score and FIB-4 score for assessing advanced fibrosis. The AUROC value for MRE, FIB-4 score, and NAFLD fibrosis score were 0.945, 0.88, and 0.86, respectively. In general, MR elastography is the best imaging modality for detecting liver fibrosis in patients with NAFLD because of its low risk of technical failure in overweight patients. However, TE and other ultrasound-based approaches may obtain unreliable results in a similar setting. The most significant limitation of MR elastography is its high cost and limited access. As a result, other imaging methods discussed here are typically attempted first. However, the optimal BMI cutoff value to decide which modality should be used first needs further investigation.

### Prospects

Non-invasive diagnoses of liver fibrosis in patients with NAFLD have made significant progress over the past decade. Given the increasing prevalence of NAFLD, this improvement is effective and promising. Serum biomarkers and developing scoring systems (the FIB-4 score, the NAFLD fibrosis score) are of increasing diagnostic and screening value for patients with NAFLD (Ngo et al., 2006; Naveau et al., 2009; Nunes et al., 2010; Parkes et al., 2010; Robic et al., 2011; Vergniol et al., 2011) and advanced fibrosis (Kim et al., 2013b; Treeprasertsuk et al., 2013).

### Future blood fibrosis biomarkers

The next-generation of functional genomic biomarkers is an emerging tool for evaluating fibrogenesis’s dynamic nature. However, validating these complex and relatively expensive methodologies is difficult, thereby limiting their clinical application. The sustained studies of the genome-wide association are a promising way to identify genomic factors and biomarkers of fibrosis (Anstee and Day, 2015). For instance, the PNPLA3 variant encoding I148M has been associated with NAFLD. However, further studies are needed to validate how these factors contribute to pathogenesis. Current proteomic research also provides several candidate serum biomarkers. Another example is represented by eicosanoid metabolites, which have been identified as potential fibrosis biomarkers (Dongiovanni et al., 2013; Anstee and Day, 2015; Dongiovanni et al., 2015).

MicroRNAs (miRNA) are small non-coding RNAs that regulate posttranscriptional gene expression and are associated with a diverse range of pathophysiologic processes (Panera et al., 2014). Several miRNAs have been proposed as potential biomarkers of progressive liver fibrosis. For instance, hepatic and serum concentrations of miRNA-122 have been associated with liver fibrosis (Miyaaki et al., 2014; Pirola et al., 2015). Identifying biomarkers based on miRNA transcripts detectable in blood or urine represents a novel approach to non-invasively diagnosing liver fibrosis and cirrhosis. However, quantifying miRNAs remains unreliable and it can produce different and varying results. Long non-coding RNAs (>200 nucleotides) and genomic and proteomic profiles of circulating extracellular vesicles may be associated with NAFLD pathogenesis. They could be used in biomarker analyses in the future. Unfortunately, compared to long non-coding RNAs (>200 nucleotides), miRNAs measured in blood or urine have not yet shown viable diagnostic or prognostic utility (Lemoinne et al., 2014; Takahashi et al., 2014).

Additionally, the application of liquid biopsy was also trialed to diagnose the presence and severity of NASH or liver fibrosis (Angelini et al., 2022). In a recent multicenter study involving 250 patients with NAFLD (Angelini et al., 2022), proteomics was performed in circulating monocytes and hepatic stellate cells, and perilipin-2 and RAB14 were measured in peripheral blood CD14^+^CD16^−^ monocytes. Results suggested that the diagnostic method based on liquid biopsy was superior to FIB4 and NAFLD fibrosis scores and that it was comparable to two-dimensional shear wave elastography. More studies on liquid biopsies to evaluate liver fibrosis should be conducted in the future.

### Future imaging methods

Elastography for detecting fibrosis in patients with NAFLD emerges as an effective method in contemporary clinical practice. However, the specific role of each test in both the clinic and investigative endeavors remains to be clarified. Moving forward, several aspects of study design should be considered.

Because of the mounting data on the effect of necroinflammatory activity and potentially hepatic steatosis on LSM, further research should define and operationalize how LSM cutoffs are interpreted in patients with variable inflammatory activity and steatosis. VCTE combined with CAP has found enough data. However, more research is needed for MRE, SWE, and ARFI to determine how to incorporate these methods into clinical practice (Petta et al., 2017). Algorithms that better calculate the effect of confounding factors could be better options.

For MRE, SWE, and ARFI, which have been increasingly applied to clinical practice in recent times, efforts must be done to provide consistent, reproducible quality criteria. MRE, SWE, and ARFI each require the operator to define the region of interest, which may cause human error and fault. As a result, research is required to determine this critical aspect of the test procedure and to formulate consensus-defined criteria that can be used to unify the quality of the selected region operator. If the regions of interest are not consistent among operators or undergo different imaging tests, the clinical meaning of any test result would be unclear (Dietrich et al., 2017).

### Future strategies for staging liver fibrosis

The rapid development of non-invasive technology has brought excellent progress in diagnosing liver fibrosis. However, it challenges researchers and brings many problems to clinical trials. Unreliable results and undeliberate clinical approaches will be a disaster for patients; that’s why liver biopsy and non-invasive methods should be used as part of an integrated system to enable more efficient and convenient management of patients with NAFLD. In addition, the combination of serum biomarkers and TE seemed to be more effective than the combination of serum biomarkers for detecting significant fibrosis, probably because diagnostic models, including liver stiffness measurements, can describe fibrotic status more completely than models merely including serum biomarkers. Further studies, such as meta-analyses and cost-effectiveness analyses that compare the accuracy and cost-saving strategies of the several biomarkers of liver fibrosis in NAFLD, both alone and in combination with imaging methods, are encouraged. Besides, artificial intelligence has provided a powerful tool to evaluate liver fibrosis in patients with NAFLD (Li et al., 2022).

Finally, this study focused on advanced fibrosis because of its high risk for hepatocellular carcinoma. It should be noted that the differentiation of NASH from simple steatosis and the identification of advanced hepatic fibrosis are critical issues in NAFLD (Castera et al., 2019However, most studies focused on significant fibrosis, advanced fibrosis, and cirrhosis, and the research for early fibrosis remained limited. Therefore, future studies should investigate the non-invasive diagnosis of early liver fibrosis.
